# Nanoengineering to Achieve High Sodium Storage: A Case Study of Carbon Coated Hierarchical Nanoporous TiO_2_ Microfibers

**DOI:** 10.1002/advs.201600013

**Published:** 2016-04-15

**Authors:** Nü Wang, Yuan Gao, Yun‐Xiao Wang, Kai Liu, Weihong Lai, Yemin Hu, Yong Zhao, Shu‐Lei Chou, Lei Jiang

**Affiliations:** ^1^ Laboratory of Bioinspired Smart Interfacial Science and Technology of the Ministry of EducationBeijing Key Laboratory of Bioinspired Energy Materials and DevicesSchool of Chemistry and EnvironmentBeihang UniversityBeijing100191P.R. China; ^2^ Institute for Superconducting and Electronic Materials (ISEM)Innovation Campus University of WollongongWollongongNSW2519Australia

**Keywords:** electrospinning, nanoporous microfibers, sodium‐ion battery, TiO_2_ anode

## Abstract

Nanoengineering of electrode materials can directly facilitate sodium ion accessibility and transport, thus enhancing electrochemical performance in sodium ion batteries. Here, highly sodium‐accessible carbon coated nanoporous TiO_2_ microfibers have been synthesised via the facile electrospinning technique which can deliver an enhanced capacity of ≈167 mAh g^−1^ after 450 cycles at current density of 50 mA g^−1^ and retain a capacity of ≈71 mAh g^−1^ at the high current rate of 1 A g^−1^. With the benefits of their porous structure, thin TiO_2_ inner walls, and the introduction of conductive carbon, the nanoporous TiO_2_/C microfibers exhibit high ion accessibility, fast Na ion transport, and fast electron transport, thereby leading to the excellent Na‐storage properties presented here. Nanostructuring is proven to be a fruitful strategy that can alleviate the reliance on materials' intrinsic nature; and the electrospinning technique is versatile and cost‐effective for the fabrication of such an effective nanoporous microfiber structure.

## 1 Introduction

With the aim of powering portable electric devices and electric vehicles, lithium‐ion batteries (LIBs) have attracted tremendous attention over the past two decades.^1^, ^2^ Intriguingly, current energy demands for large‐scale energy storage have intensified the pursuit of sodium ion batteries (SIBs) because of their low price and the natural abundance of sodium.^3^, ^4^, ^5^ Many electrode materials for LIBs have correspondingly been investigated for SIBs, based on their chemical similarities.^6^ In contrast to Li ions (0.76 Å), the larger Na ions (1.02 Å) tend to show sluggish kinetics for electrochemical reactions, which generally results in unsatisfactory battery performance or even complete electrochemical inactivity.^7^, ^8^, ^9^, ^10^ A common and effective strategy to improve the kinetics is to fabricate nanostructured materials,^11^, ^12^ which not only can shorten the ions' diffusion paths but also enlarges the electrode/electrolyte contact area. Nanostructured electrodes are also favourable for alleviating mechanical stress and maintaining the structural integrity of the electrode. For instance, expanded graphite delivers a surprisingly high capacity of 284 mAh g^−1^ via nanostructural control of inactive graphite (less than 35 mAh g^−1^) in SIBs.^13^ Another functional strategy is to enhance the conductivity of electrodes via doping modification or incorporating conductive materials.^14^, ^15^, ^16^ Typically, these two strategies can synergistically alleviate the limitations of the materials' intrinsic natures and greatly optimize the sodium‐storage properties of electrode materials.^17^ Impressively, both strategies have been extensively applied into anode materials design and reach significant achievement, such as nanosized Li_4_Ti_5_O_12_,^18^ Sn nanoparticles embedded in C,^19^ yolk‐shell Sn_4_P_3_@C nanospheres,^20^ yolk‐shell FeS@C nanospheres,^21^ and phosphorene‐graphene hybrid.^22^ For instance, in contrast to the steep capacity decline found in commercial MoS_2_, the electrochemical performance in terms of capacity and cycling stability can be significantly enhanced by constructing MoS_2_/graphene nanocomposites.^23^

Low‐conductivity anatase TiO_2_ was selected to demonstrate the process for accelerating the Na‐reaction kinetics. TiO_2_ has been intensively investigated as a potential anode material for SIBs owing to its many merits in terms of its cycling stability, nontoxicity, low cost, and abundance.^24^, ^25^, ^26^, ^27^, ^28^, ^29^ Amorphous TiO_2_ nanotubes grown on a Ti substrate delivered a gradually increasing capacity of up to 120 mAh g^−1^ at 50 mAg^−1^.^24^ A thin carbon layer coated on anatase TiO_2_ nanorods was proven to effectively enhance the rate capability. In addition, it was proposed that the reaction mechanism involves Na ion insertion to form orthorhombic Na_0.5_TiO_2_.^28^ Most recently, via a special microwave synthesizer, Huang and co‐workers' reported the Na^+^ intercalation pseudocapacitance of graphene‐coupled TiO_2_, which realized a specific capacity of 90 mAh g^−1^ at 12 A g^−1^.^27^ Superb amorphous TiO_2_ stands out in LIBs and SIBs due to its intrinsically isotropic nature, which is believed to possess open active diffusion channels, initially facilitating Li and Na‐ion mobility, ionic and electronic diffusion into TiO_2,_ leading to high ion accessibility and enhanced power and energy densities.^28^, ^30^ Based on these productive advances, it is necessary to further develop a simple method to fabricate ideal hierarchical nanostructured TiO_2_‐based composites with low cost and high productivity.

In this work, we have designed and fabricated hierarchical nanoporous TiO_2_‐carbon microfibers (NTMFs‐C) with large surface area and enhanced conductivity. The highly nanoporous structure can be formed by a straightforward, general microemulsion electrospinning method with subsequent calcination. This synthesis technique does not require expensive facilities and tedious preparation processes. The obtained NTMF‐C microfibers have continuous 1D geometry with hierarchical nanopores inside, which is able to support fast electron transport and guarantee high ion accessibility. The multilevel nanoporous TiO_2_‐C microfibers are expected to show long‐term cycling stability and enhanced rate capability with high specific capacity. The electrode delivers a high capacity of ≈167 mAh g^−1^ over 450 cycles at current density of 50 mA g^−1^ and achieves a capacity of ≈71 mAh g^−1^, even at the high current rate of 1 A g^−1^. Our results confirm the crucial functions of such a nano‐engineering strategy toward optimization of electrode performance, which will arouse further attention and offer a versatile technique for fabricating active materials, especially regarding those with insufficient intrinsic properties.

## 2 Results and Discussion

**Figure 1:**
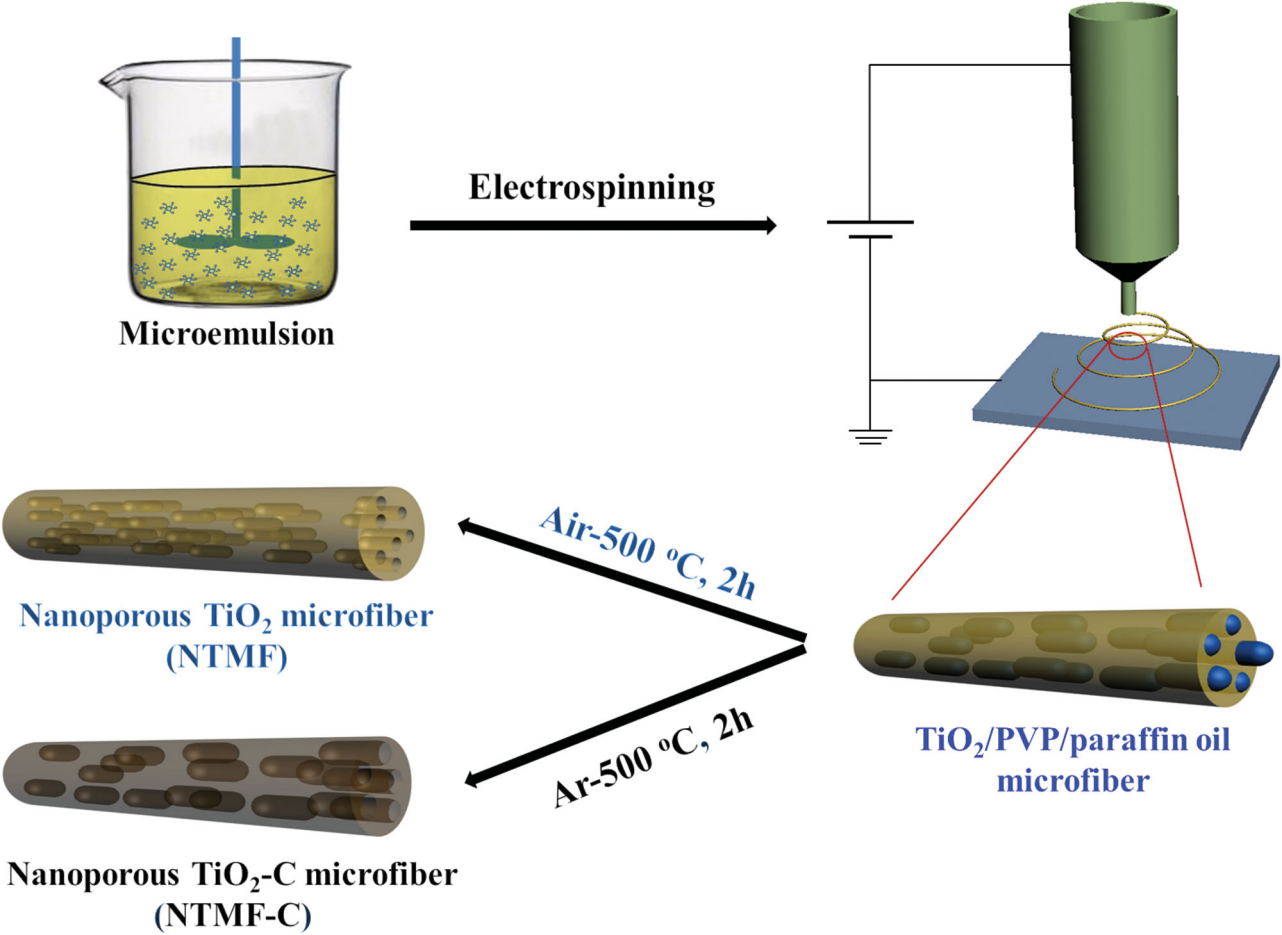
Schematic illustration of the synthetic processes for producing nanoporous TiO_2_ and TiO_2_‐C microfibers via electrospinning.

**Figure 2:**
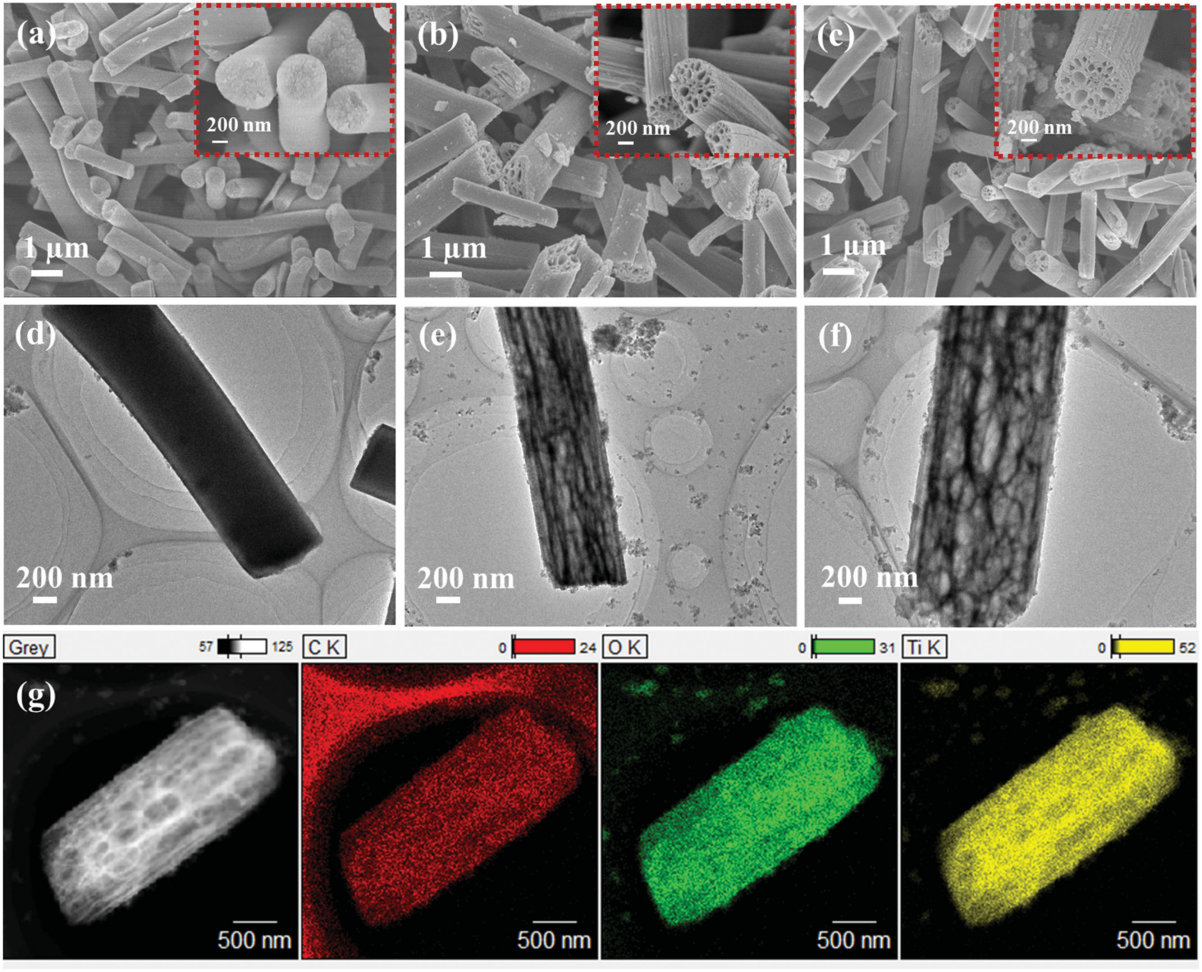
SEM images of a) STMF, b) NTMF, and c) NTMF‐C, with corresponding SEM images at higher magnification (insets). TEM images of d) STMF, e) NTMF, and f) NTMF‐C for single microfibers. g) Elemental mapping of NTMF‐C for C, O, and Ti elements.

The synthetic procedures for hierarchical NTMF‐C and nanoporous TiO_2_ microfibers (NTMF) are illustrated in **Figure** **1**. The as‐prepared microemulsion was electrospun to form microfibers in a high electric field. Two samples, with and without carbon, could be separately fabricated by simply varying the calcination atmosphere. Under air atmosphere, the random nanosized pores are created from vacancies left by oil droplets and combustion of PVP in the microemulsion. When calcined under Ar atmosphere, the morphology of the nanopores does not only depend on the oil drops, but also on the residual carbon matrix from PVP. NTMF‐C and NTMF, therefore, likely possess distinguishable configurations in their morphologies. This speculation is confirmed by the TEM images in **Figure** **2**.

The scanning electron microscopy (SEM) images in Figure 2a and the transmission electron microscopy (TEM) image in Figure 2d reveal that the as‐prepared solid TiO_2_ microfibers (STMF) possess smooth surfaces and has a cylindrical solid structure. The SEM image at higher magnification (inset of Figure 2a) confirms that the obtained microfibers of the STMF sample are solid and uniform, and an average diameter of ≈632 nm was obtained (Figure S1, Supporting Information). The NTMF and NTMF‐C obtained by microemulsion electrospinning show similar morphologies, in which there are massive nanopores inside each microfiber (Figure 2b,c). The high quality nanostructure of the homogeneous nanoporous microfibers can be observed in the SEM inset images. The optical photographs of the NTMF (white) and NTMF‐C (black) microfibers confirm the successful incorporation of carbon (Figure S2, Supporting Information). The morphological differences of all the samples were examined in detail using transmission electron microscopy (TEM) analysis. Unlike the STMF, the NTMF and NTMF‐C samples manifest very high porosity. It is interesting that the NTMF sample shows tube‐like pores (Figure 2e), while NTMF‐C displays a bubble‐like structure (Figure 2f), which is mainly ascribed to the confinement effect of the retained carbon matrix. More importantly, NTMF‐C and NTMF show very thin wall thicknesses, which correspond to shortened Na ion transport paths. The energy‐dispersive spectroscopy (EDS) elemental mapping confirmed the coexistence and homogeneous dispersion of the C, O, and Ti elements throughout the nanoporous TiO_2_/C microfibers (Figure 2g).

**Figure 3:**
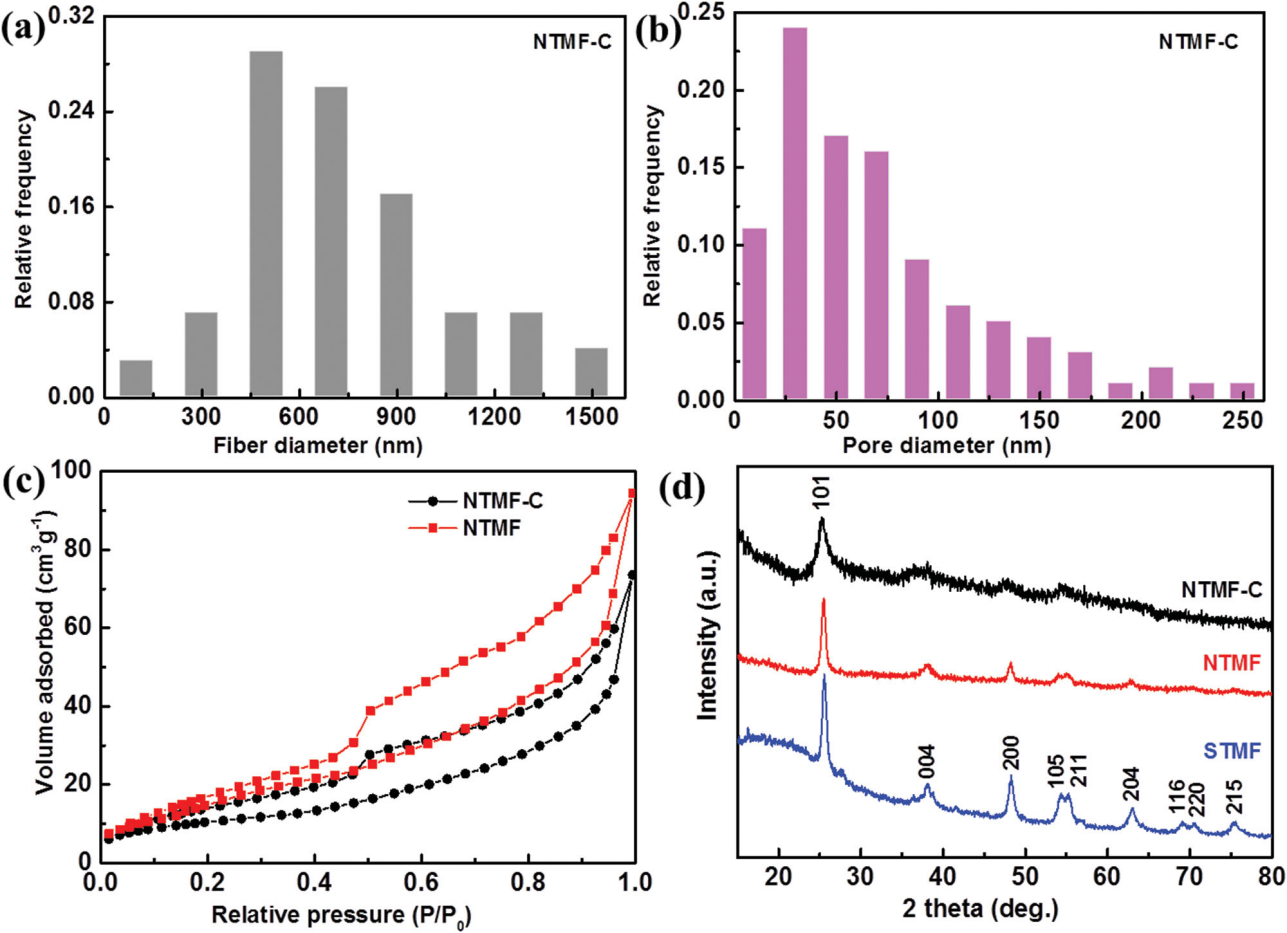
a) Fiber diameter distribution, and b) pore diameter distribution of NTMF‐C sample. c) Nitrogen sorption isotherms of NTMF‐C and NTMF. d) XRD patterns of NTMF‐C, NTMF, and STMF.

As shown in **Figure** **3**a,b, it is noteworthy that the bubble‐like NTMF‐C shows a higher average fiber diameter of ≈720 nm and a larger pore diameter of ≈72 nm, which corresponds to ≈630 and ≈60 nm for the tube‐like NTMF (Figure S3, Supporting Information), respectively. The high‐porosity and larger‐diameter NTMF‐C microfibers are believed to contain a larger amount of electrolyte per microfiber, so a larger number of Na ions are available to establish the critical ion concentration for cycling. The carbon ratio (ω_C_) in the composite is estimated to be ≈29 wt% (Figure S4, Supporting Information). Nitrogen sorption analysis of NTMF and NTMF‐C shows type‐IV curves with H4 hysteresis (Figure 3c), demonstrating the disordered distribution of nanopores. The Brunauer–Emmett–Teller (BET) surface areas were calculated to be 67 and 38 m^2^·g^−1^ for NTMF and NTMF‐C, respectively. Figure 3d shows the X‐ray diffraction (XRD) patterns of the STMF, NTMF, and NTMF‐C samples. All XRD patterns could be indexed to a typical TiO_2_ anatase phase with space group I41/amd (JCPDS No. 21‐1272). No peaks from other phases can be detected, indicating the high purity of the obtained samples. Compared with the STMF, the NTMF shows lower peak intensity with the absence of some phases, which indicates a lower degree of TiO_2_ crystallization. Moreover, the NTMF‐C pattern indicates obvious amorphous properties, only showing broadened (101), (004), and (200) humps, implying small crystals of TiO_2_ formed with the aid of the C matrix. The unique nanoporous TiO_2_‐C microfibers, featuring large nanopores, high conductivity, and thin inner walls, were expected to enhance the availability of ions near the surface and facilitate efficient electron and Na ion transport. This nanostructure, in turn, is expected to yield enhanced capacity, long‐term stability, and excellent rate capability.

**Figure 4:**
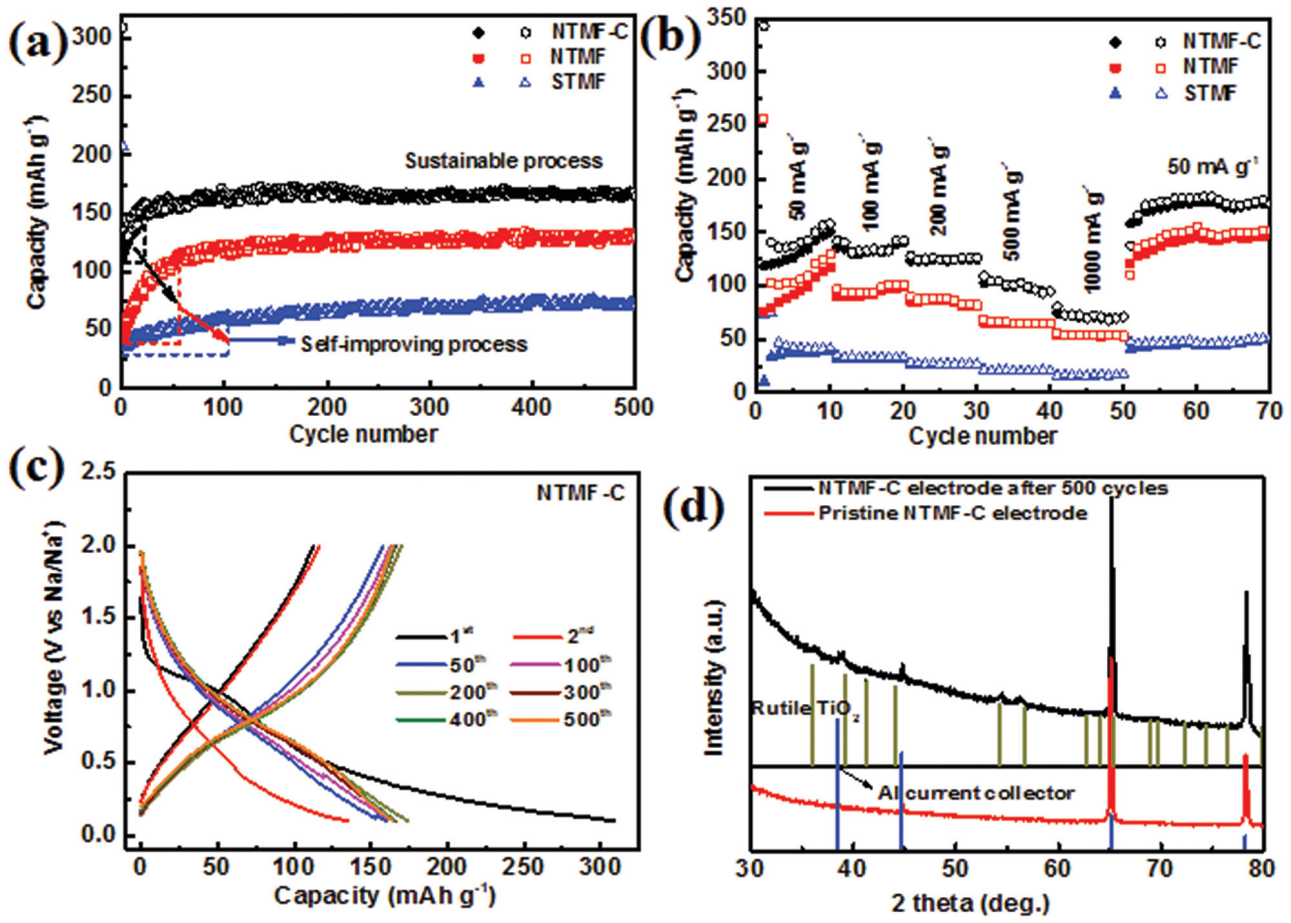
a) Cycling performance, b) rate capability of NTMF‐C, NTMF, and STMF; c) charge/discharge curves at selected cycles of NTMF‐C, and d) ex situ XRD of NTMF‐C before and after 500 cycles.

As illustrated in **Figure** **4**a, it is interesting that all three electrodes show self‐improving capacity, which is mostly due to the occurrence of an irreversible phase transition from amorphous TiO_2_ to cubic phase. The newly formed cubic phase can exhibit self‐improving capacity via self‐organization based on the electrochemical reactions between TiO_2_ and sodium ions.^23^ The solid TiO_2_ microfibers (STMF) undergo slow self‐organization over 100 cycles due to their good crystallization, which leads to a slight capacity increase from 41 to 60 mA h g^−1^. It can be seen that the nanoporous TiO_2_ microfibers (NTMF) complete the self‐improving process after about 50 cycles, which corresponds to a great capacity increase of 67 mA h g^−1^ (from 40 to 107 mA h g^−1^). Afterwards, the NTMF delivers outstanding cycling stability, with a capacity of ≈130 mA h g^−1^ over 500 cycles (sustainable process). In contrast, the porous NTMF‐C hybrid shows the most rapid self‐improving process and the highest specific capacity. A high and fast self‐improving capacity to ≈150 mA h g^−1^ can be achieved after 20 cycles, with a sustainable capacity of 167 mA h g^−1^ over the subsequent 500 cycles. Significantly, the practical capacity of NTMF‐C is estimated to be ≈116 mA h g^−1^ based on the following calculation: Q_NTMF‐C_ = Q_TiO2_ × (1‐ω_C_) + Q_C_ × ω_C_ = 130 × 0.71 + 82 × 0.29 = 116.08 mA h g^−1^ (Q_TiO2_: the practical capacity of pure NTMF; Q_C_: the practical capacity of nanoporous carbon microfibers, see Figure S5 in the Supporting Information). It is noteworthy that the sustainable capacity (167 mA h g^−1^) of NTMF‐C is much higher than the estimated practical capacity, which means the introduction of C accounts for the enhancement of electrochemical activity of TiO_2_ with Na^+^. As shown in Figure 4b, with the aid of nanoporosity and carbon incorporation, the specific capacity of NTMF‐C is superior at the various current rates compared with STMF and NTMF‐C. Both the NTMF‐C and the NTMF electrodes show an obvious self‐improving phenomenon when initially cycled at 50 mA g^−1^ and can reach a capacity of ≈120 and 150 mA h g^−1^, respectively. Clearly, the NTMF‐C electrode shows slower capacity decay when the current rates increase. The retained capacity at 1000 mA g^−1^ is 71 and 50 mA h g^−1^ for NTMF‐C and NTMF, corresponding to capacity retention of 47% and 41.6%, respectively. With the current density back to 50 mA g^−1^, an impressive reversible capacity of ≈180 mA h g^−1^ is restored for NTMF‐C electrode, in contrast to ≈150 mA h g^−1^ for NTMF electrode. The STMF, however, delivers negligible capacity at high current rates. In conclusion, NTMF‐C exhibits the best Na‐storage properties in term of reversible capacity and rate capability, indicating the effective functions of the nanoporous structure and C incorporation toward maximizing the electrochemical reactions between TiO_2_ and Na^+^. The Na‐storage mechanism of TiO_2_ microfibers is investigated via charge/discharge curves and ex situ XRD of NTMF‐C electrode after 500 cycles, since the STMF and NTMF show similar charge/discharge profiles to NTMF‐C (Figure S6, Supporting Information). As shown in Figure 4c, with TiO_2_ phase transition over cycling, the electrode could rapidly reach high and stable capacity with slight‐plateau profiles at both charge and discharge state, which corresponds to the Na^+^ insertion/extraction coupled with Ti^4+^/^3+^ redox reaction, similar to that rutile TiO_2_ and nanostructured rutile studied in LIBs.^31^, ^32^ Moreover, in contrast to the pristine NTMF‐C electrode, four conspicuous peaks occur at 36.05°, 39.1°, 54.3°, and 56.5°, which can be indexed to be rutile TiO_2_ (JCPDS No. 21‐1276). These results prove that the amorphous TiO_2_ is able to transit into rutile phase after repeated cycles, which is responsible for the enhanced capacity and rate capability.

**Figure 5:**
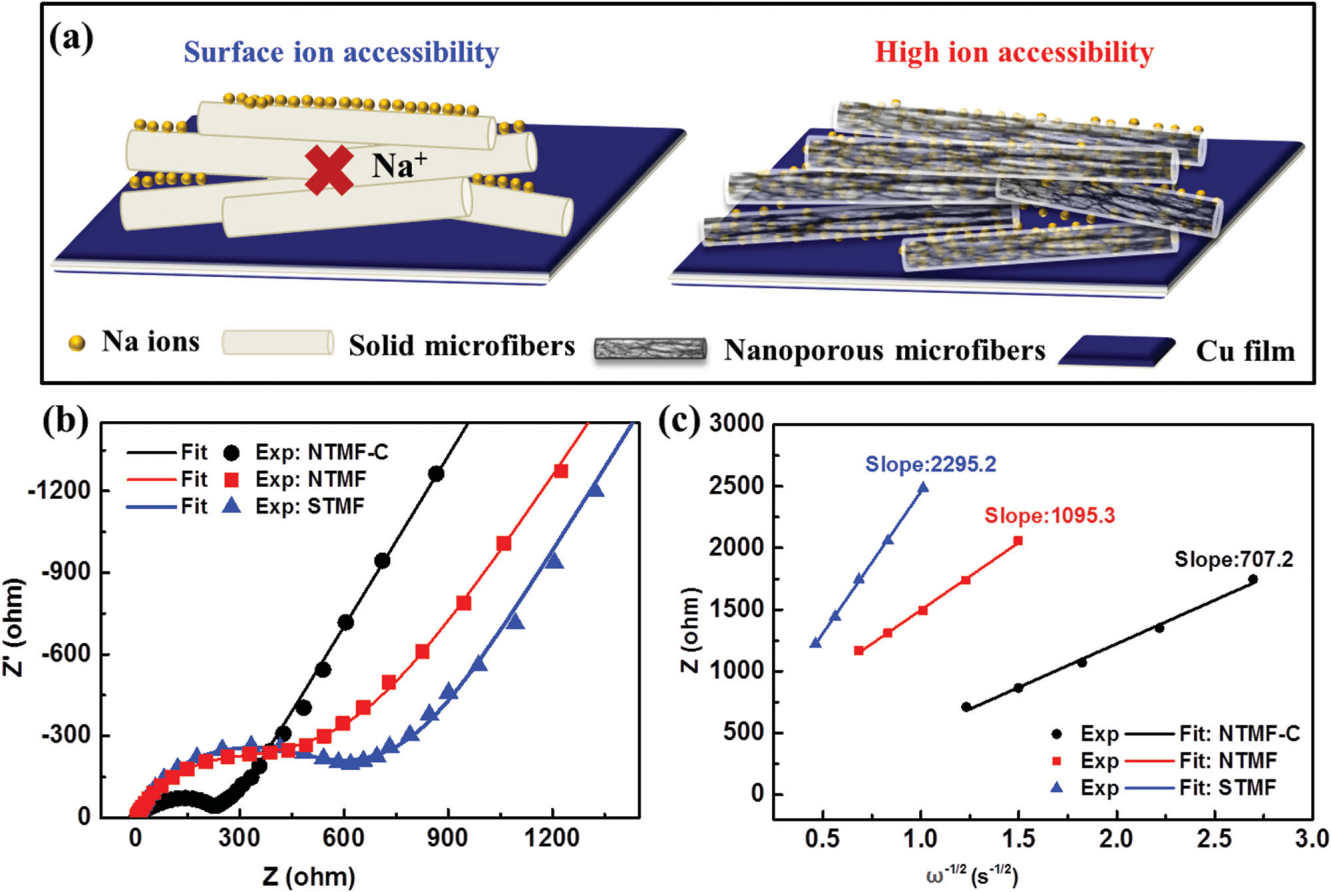
a) Schematic illustration of ion accessibility for the solid microfibers and nanoporous microfibers during sodiation/desodiation processes. b) Impedance plots for the three anodes after rate capability testing at frequencies from 100 kHz to 10 mHz. c) Fitting lines for the real part of the complex impedance versus ω^−1/2^ at 25 °C.

As shown in **Figure** **5**a, the inferior performance of STMF is mostly due to the limited ion accessibility and sluggish Na^+^ ion diffusion through the bulk fibers. When nanopores are created in the TiO_2_ microfibers, the obtained NTMF is expected to allow high concentrations of Na^+^ ions to be absorbed in the nanopores and efficiently initiate the phase transition reaction. The superior electrochemical performance of NTMF‐C is partially ascribed to the high degree of amorphization, which leads to rapid occurrence of the self‐improving process. On the other hand, the presence of a huge density of nanopores could provide a high amount of active TiO_2_ surface, a large TiO_2_/electrolyte contact area, and short diffusion paths for electron and ions. This nanoporous structure, therefore, facilitates the transport of ions to the TiO_2_ host. Furthermore, large‐diameter pores can accommodate a large volume of electrolyte, through which abundant Na ions can be used to establish the critical ion concentration that supports electrochemical cycling. Thus, the availability of ions near the TiO_2_ host is very much responsible for the observed improvement of the capacity over cycling. In addition, the enhanced conductivity of the electrode can be attributed to the residual C, which guarantees fast electron and sodium ion transportation as well.

In order to confirm the enhanced kinetic processes of the NTMF‐C, electrochemical impedance spectroscopy (EIS) of all samples was conducted after rate capability testing at 25 °C. As shown in Figure 5b, the Nyquist plots of the three electrodes were collected from 0.1 mHz to 1 MHz. All the impedance curves show typical semicircles in the high to medium frequency region, which are associated with the solid electrolyte interphase (SEI) resistance (*R*_SEI_) and the charge transfer resistance (*R*_ct_). According to the fitting results, the NTMF‐C electrode has a very low *R*_SEI_ of 21.3 Ω, in sharp contrast to 376.2 Ω and 427.8 Ω for the NTMF and STMF electrodes, respectively, indicating that a favorable SEI film was formed in the NTMF‐C electrode. It is believed that *R*_ct_ is affected by the sodium ion transport and the conductivity of the electrodes. Similarly, the NTMF‐C electrode shows a low *R*_ct_ (176.6 Ω) as well, which is 530 and 634 Ω for the NTMF and STMF electrodes, respectively. The significantly low *R*_ct_ value of the hybrid implies fast kinetic processes, consistent with the superior electrochemical performance in Figure 4. The linear part of the EIS spectrum with an angle of ≈45° in the low frequency region corresponds to the Warburg impedance (*W*), associated with the sodium‐ion diffusion in the TiO_2_ host. Based on the calculation of the lithium‐ion diffusion coefficient,^33^, ^34^, ^35^ the sodium‐ion diffusion coefficient could also be estimated via the following equation^36^

(1) $$D=R^{2}T^{2}/2A^{2}n^{4}F^{4}C^{2}\sigma^{2}$$

where *D* is the diffusion coefficient (cm^2^·s^−1^), *R* is the gas constant (8.314 J mol^−1^ K^−1^), *T* is the absolute temperature (25 °C), *A* is the surface area of the anode (0.724 cm^2^), *n* is the number of electrons transferred in the half‐reaction for the redox couple (0.5), *F* is the Faraday constant (96 500), *C* is the concentration of Na ions in the solid material (assuming the same value as its Li counterpart: 1.39 × 10^−3^ mol cm^−3^), and *σ* is the Warburg factor. As shown in Figure 5b, *σ* can be obtained from the linear slope of Z versus *ω* ^−1/2^. The sodium‐ion diffusion coefficients are calculated to be 7.5 × 10^−15^, 3.1 × 10^−15^, and 7.1 × 10^−16^ cm^2^ s^−1^ for NTMF‐C, NTMF, and STMF, respectively. As expected, this result confirms that the fastest sodium ion transportation rate occurs in the nanoporous TiO_2_‐C microfiber electrode. The diffusion time, *t*, for Na ions passing through the TiO_2_ host is calculated via the following equation

(2) $$t=L^{2}/D$$

in which *L* is the diffusion path and *D* is the above‐calculated diffusion rate. For STMF, *L* is the average radius of all fibers (≈312.5 nm). For NTMF and NTMF‐C, *L* is the average wall thickness of the nanopores (≈18 nm). *t* is calculated to be 4.3 s, 105 s, and 13754 s for NTMF‐C, NTMF, and STMF, respectively.

## 3 Conclusions

In summary, in order to accelerate the sodium kinetics of TiO_2_ anode, highly nanoporous TiO_2_‐C hybrid microfibers were successfully synthesized via a simple and scalable electrospinning strategy. The obtained microfibers demonstrate interesting and outstanding electrochemical performance, undergoing self‐improving and sustainable processes. The TiO_2_‐C electrode delivers a capacity increase of ≈50 mAh g^−1^ over 20 cycles, retaining capacity of ≈167 mAh g^−1^ after 450 cycles at current density of 50 mA g^−1^, and a capacity of ≈71 mAh g^−1^ at the high current rate of 1 A g^−1^, with evident self‐improving capacity when cycled back to the initial low rate. The nanoporous structure with thin inner walls is able to realize high ion accessibility and fast ion transport. The incorporation of carbon can guarantee fast ion transport. The enhanced performance confirms the essential functions of the electrode configuration. This unique strategy for nanoporous microfiber construction is universally applicable and easy to implement, and it is expected to be extended to other electrode materials, especially transition metal oxides.

## 4 Experimental Section

*Preparation of Nanoporous TiO_2_‐Carbon Microfibers, Nanoporous TiO_2_ Microfibers, and Solid TiO_2_ Microfibers*: 0.5 g polyvinyl pyrrolidone (PVP, Mw ≈ 1 300 000) and 0.25 g hexadecyl trimethyl ammonium bromide (CTAB) were first dissolved in a mixture of 0.5 g acetic acid and 6.5 g ethanol. Then, 3.0 g tetrabutyl titanate (Ti(OBu)_4_ was added slowly, followed by stirring for 2 h. Afterward, 1.5 g paraffin oil was further added to form a homogeneous and transparent microemulsion after stirring for another 2 h.^32^ The obtained microemulsion was loaded into a 5 mL syringe with a blunt‐ended metallic needle. A syringe pump was used to control the flow rate of the jetting solution (0.6–1 mL h^−1^). The needle was connected to a high voltage power supply, and a stainless steel plate covered by a piece of aluminium foil was used as the collector. The working distance between the needle tip and the collector was 20 cm, and the voltage was 15–25 kV. Finally, the TiO_2_‐carbon microfibers (NTMF‐C) and nanoporous TiO_2_ microfibers (NTMF) were obtained after calcination at 500 °C in Ar and O_2_ atmosphere for 2 h with a heating rate of 5 °C min^−1^, respectively. For comparison, solid TiO_2_ microfibers (STMF) were obtained by a similar electrospinning process. The electrospinning solution was prepared as follows. 1 g PVP was dissolved in a mixture of 0.5 g acetic acid and 5 g ethanol, followed by adding 3.0 g Ti(OBu)_4_ and stirring for 2 h.

*Structural Characterizations*: The morphologies of the samples were investigated by field‐emission scanning electron microscopy (SEM, JEOL JSM‐7500) and transmission electron microscopy (TEM, JEOL ARM‐200F) operated at an acceleration voltage of 200 kV. The porosity of the NTMF‐C and NTMF samples was measured by nitrogen sorption isotherms at 77 K under relative pressure (*P*/*P*_0_) from 0.04 to 0.2 with a Micromeritics Tristar 3020 analyzer (USA). Before measurements, the samples were degassed in vacuum at 180 °C for at least 6 h. The BET method was utilized to calculate the surface areas. The crystal structure and phase of products were characterized using powder X‐ray diffraction (XRD; GBC MMA diffractometer) with Cu Kα radiation at a scan rate of 2 min^−1^. The thermal decomposition behavior of the products was monitored by using by using thermogravimetric analysis (TGA, PerkinElmer TG/DTA 6300) from 30 to 900 °C in air with a heating rate of 5 °C min^−1^.

*Electrochemical Measurements*: Measurements of the sodium‐storage properties were conducted on coin‐type half cells assembled in an argon‐filled glove box. The electrode slurry was prepared by mixing 70 wt% active material (NTMF‐C, NTMF, and STMF), 20 wt% Super P, and 10 wt% carboxymethyl cellulose (CMC) in a planetary mixer (KK‐250S). The electrode films were prepared by pasting the slurry on copper foil using a doctor blade in a thickness of 100 μm, followed by drying in a vacuum oven at 80 °C overnight. The working electrodes were obtained by punching the above electrode film into discs 0.96 cm in diameter. Sodium foil was used as both reference and counter electrode. The electrodes were separated by glass fiber separators. The electrolyte was prepared with 1.0 m NaClO_4_ in 1:1 (volume ratio) ethylene carbonate (EC)/propylene carbonate (PC) with 5 wt% fluoroethylene carbonate (FEC) additive. The electrochemical performances were investigated on a Land Battery Tester with a cut‐off voltage range from 0. 1 to 2.0 V (vs Na/Na^+^). Impedance testing was performed using a Biologic VMP‐3 electrochemical workstation.

## Supporting information

As a service to our authors and readers, this journal provides supporting information supplied by the authors. Such materials are peer reviewed and may be re‐organized for online delivery, but are not copy‐edited or typeset. Technical support issues arising from supporting information (other than missing files) should be addressed to the authors.

Supplementary
