# Supplementary material for: Nanoengineering to Achieve High Sodium Storage: A Case Study of Carbon Coated Hierarchical Nanoporous TiO2 Microfibers
Source: Adv Sci (Weinh). 2016 Apr 15;3(8):1600013. doi: 10.1002/advs.201600013 (PMC5074262; doi:10.1002/advs.201600013)
Supplement: Supplementary file 1 — Supplementary [file ADVS-3-0a-s001.pdf]

Copyright WILEY-VCH Verlag GmbH & Co. KGaA, 69469 Weinheim, Germany, 2016.

Supporting Information

for *Adv. Sci.,* DOI: 10.1002/advs.201600013

Nanoengineering to Achieve High Sodium Storage: A Case Study of Carbon Coated Hierarchical Nanoporous TiO_2 Microfibers

Nü Wang, Yuan Gao, Yun-Xiao Wang,* Kai Liu, Weihong Lai, Yemin Hu, Yong Zhao, Shu-Lei Chou,* and Lei Jiang*

Supporting information

Nanoengineering to achieve high sodium storage: A case study of carbon coated hierarchical nanoporous TiO_2 microfibers

Nü Wang, [†,‡,*] *Yuan Gao,*[†] *Yun-Xiao Wang,* [‡,*] *Kai Liu,*[†] *Weihong Lai,*[‡] *Yemin Hu,*[‡] *Yong Zhao,*[†] *Shu-Lei Chou,* [‡,*] *Lei Jiang*[†]

Dr. N Wang, Y. Gao, K. Liu, Prof. Y. Zhao, Prof. L. Jiang

† Laboratory of Bioinspired Smart Interfacial Science and Technology of the Ministry of Education, Beijing Key Laboratory of Bioinspired Energy Materials and Devices, School of Chemistry and Environment, Beihang University, Beijing 100191, China.

* E-mail: wangn@buaa.edu.cn

Dr. Y.-X. Wang, W. Lai, Dr. Y. Hu, Dr. S.-L. Chou

‡Institute for Superconducting & Electronic Materials (ISEM), Innovation Campus University of Wollongong, Wollongong, NSW, 2519, Australia.

* E-mail: yunxiao@uow.edu.au; shulei@uow.edu.au

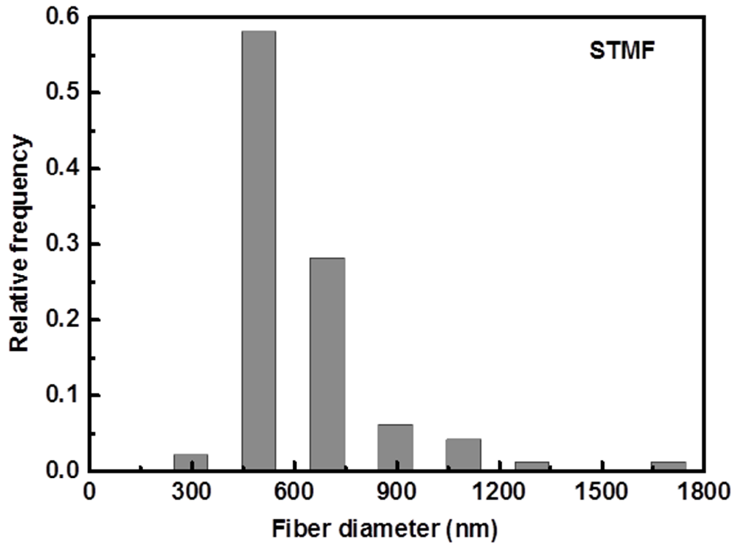

Figure S1. Fiber diameter distribution of solid TiO_2 microfibers (STMF).

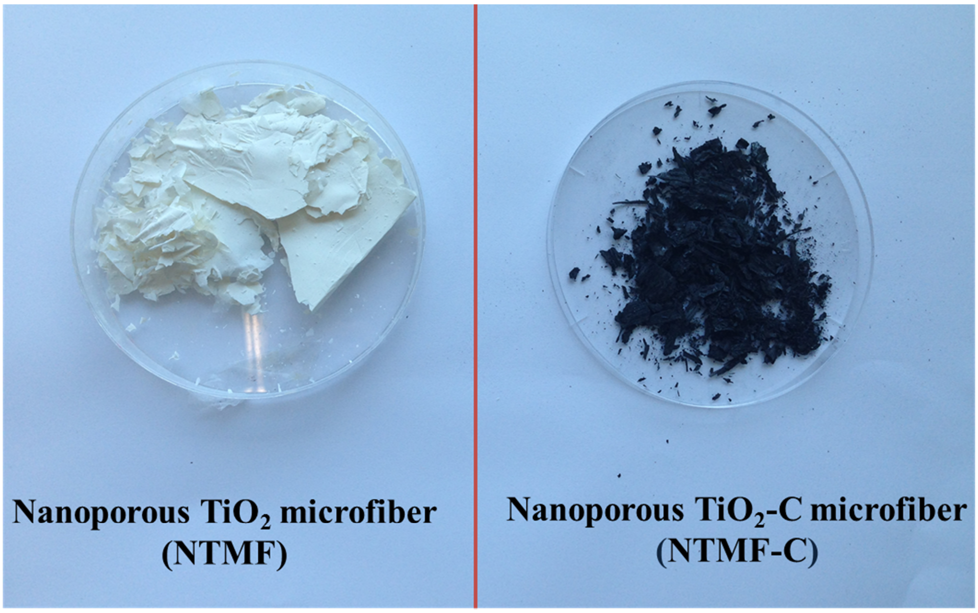

Figure S2. The optical photographs of the NTMF (white) and NTMF-C (black) microfibers.

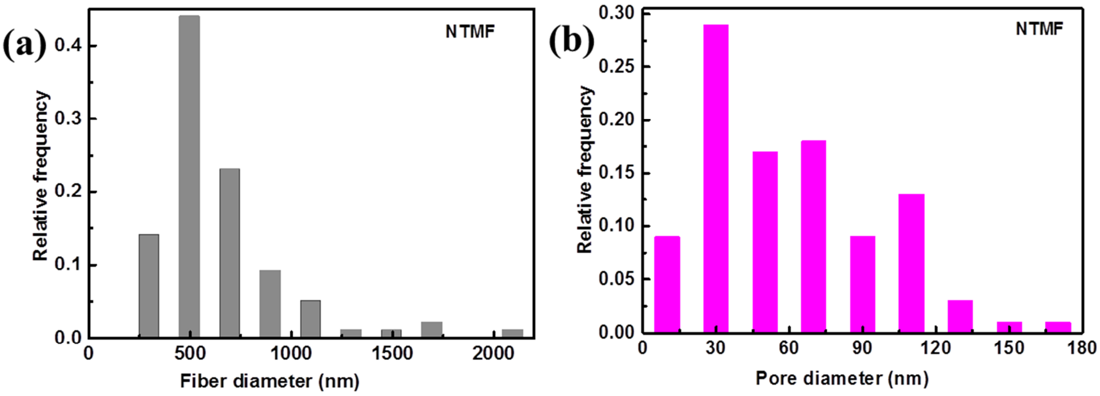

Figure S3. (a) Fiber diameter distribution, and (b) pore diameter distribution of nanoporous TiO_2 microfiber (NTMF) sample.

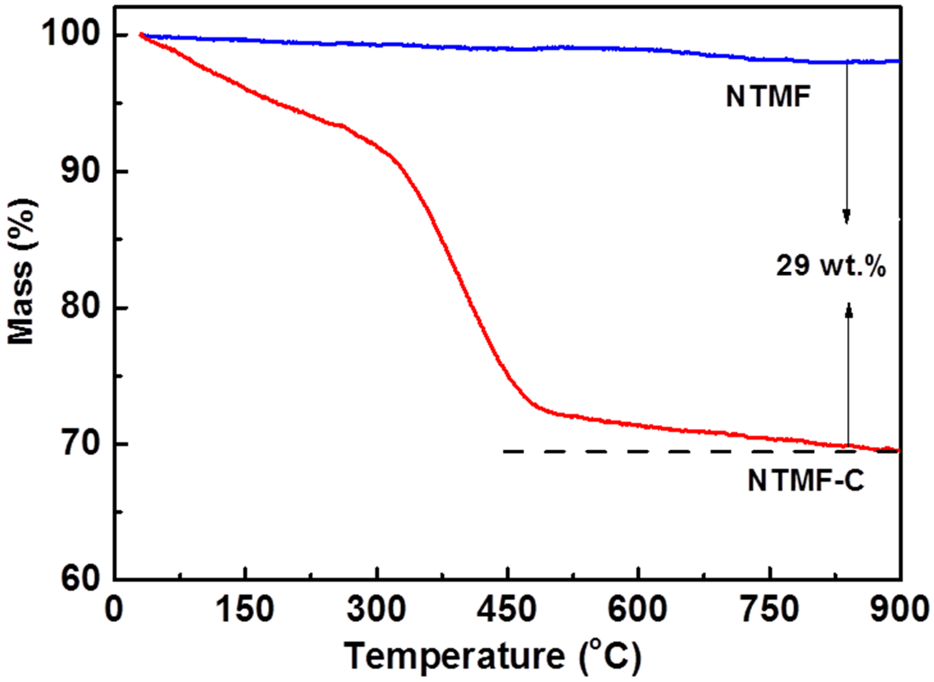

Figure S4. TGA curves of NTMF and NTMF-C samples.

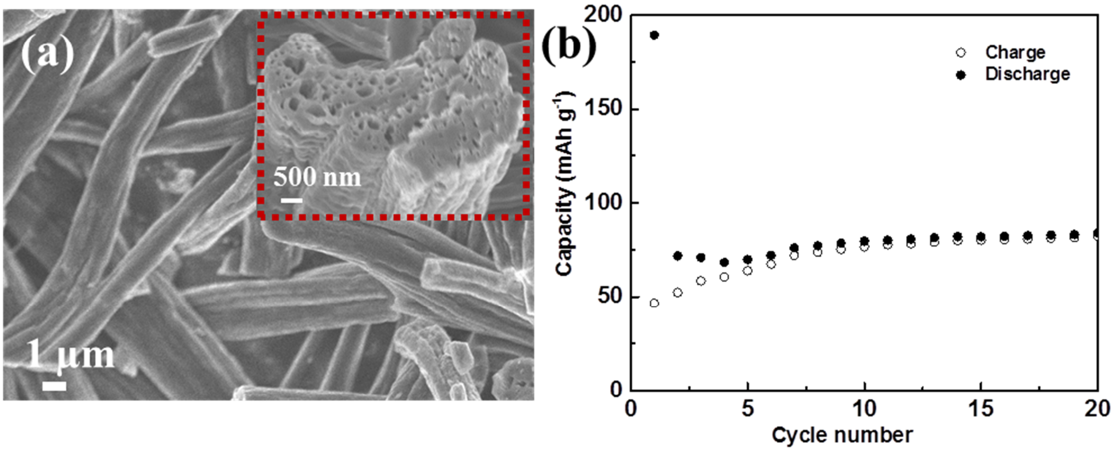

Figure S5. (a) SEM images of nanoporous carbon microfibers with corresponding SEM images at higher magnification (insets). (b) Cycling performance of mesoporous carbon microfibers in SIBs.

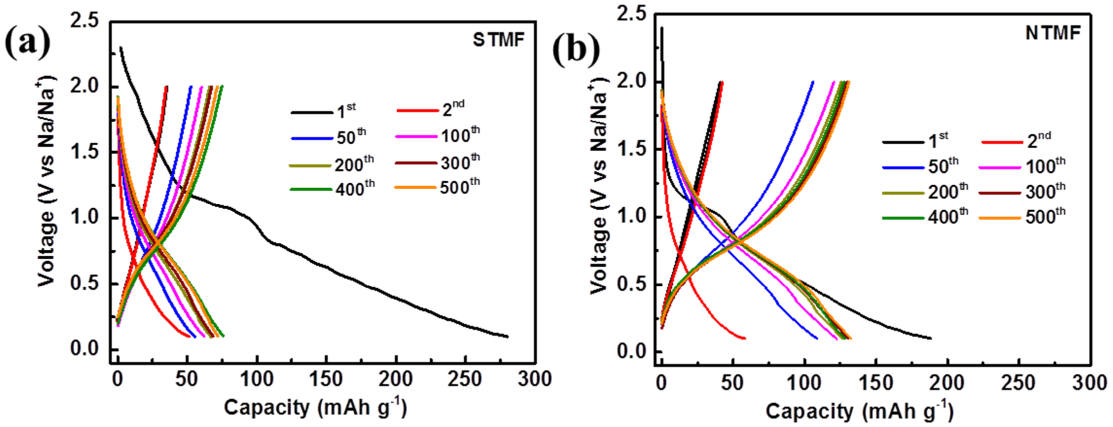

Figure S6. Charge/discharge profiles of (a) STMF and (b) NTMF at selected cycles.
